# Detection of *mcr-1* Gene in Undefined *Vibrio* Species Isolated from Clams

**DOI:** 10.3390/microorganisms10020394

**Published:** 2022-02-08

**Authors:** Christian Valdez, Cátia Costa, Marco Simões, Carla C. C. R. de Carvalho, Teresa Baptista, Maria J. Campos

**Affiliations:** 1 MARE-Marine and Environmental Sciences Centre, ESTM, Polytechnic of Leiria, 2520-630 Peniche, Portugal; 4180268@my.ipleiria.pt (C.V.); catia.g.costa@ipleiria.pt (C.C.); marco.a.simoes@ipleiria.pt (M.S.); teresa.baptista@ipleiria.pt (T.B.); 2 iBB-Institute for Bioengineering and Biosciences, Department of Bioengineering, Associate Laboratory i4HB—Institute for Health and Bioeconomy, Instituto Superior Técnico, Universidade de Lisboa, Av. Rovisco Pais, 1049-001 Lisboa, Portugal; ccarvalho@tecnico.ulisboa.pt

**Keywords:** antimicrobial resistance, colistin, *mcr-1*, *Vibrio* spp.

## Abstract

The increase of antimicrobial resistant strains is leading to an emerging threat to public health. Pathogenic *Vibrio* are responsible for human and animal illness. The Enterobacteriaceae family includes microorganisms that affect humans, causing several infections. One of the main causes of human infection is related to the ingestion of undercooked seafood. Due to their filter-feeding habit, marine invertebrates, such as clams, are known to be a natural reservoir of specific microbial communities. In the present study, Vibrionaceae and coliforms microorganisms were isolated from clams. A microbial susceptibility test was performed using the disk diffusion method. From 43 presumptive *Vibrio* spp. and 17 coliforms, three *Vibrio* spp. with MICs to colistin >512 mg L^−1^ were found. From the 23 antimicrobial resistance genes investigated, only the three isolates that showed phenotypic resistance to colistin contained the *mcr-1* gene. Genotypic analysis for virulence genes in EB07V indicated *chiA* gene presence. The results from the plasmid cure and transformation showed that the resistance is chromosomally mediated. Biochemical analysis and MLSA, on the basis of four protein-coding gene sequences (*recA, rpoB*, *groEL* and *dnaJ*), grouped the isolates into the genus *Vibrio* but distinguished them as different from any known *Vibrio* spp.

## 1. Introduction

The genus *Vibrio* contains more than 130 species [1,2] and includes both non-pathogenic and pathogenic species, being highly genetically diverse [3,4]. *Vibrio* spp. are Gram-negative motile bacteria, facultative anaerobic, and non-spore-forming. With cells shaped as curved or straight rods, and typically found in freshwater, estuarine and marine environments [1,5]. Pathogenic species are associated with several human diseases caused by the natural microbiota of aquatic environments [6] and are mostly linked to the ingestion of undercooked seafood [7]. Vibriosis is one of the most prevalent bacterial diseases associated with marine fish and shellfish [8]. Non-pathogenic *Vibrio*, on the other hand, are not associated with human illnesses, and are, therefore, not routinely identified [9]. These so-called “marine species” or “marine vibrios”, have nonetheless been frequently isolated, thus receiving more attention in recent years since they have an important role in specific ecological niches and belong to autochthonous marine microbial communities [4]. These niches are often fish farms, where antibiotics are frequently used, and *Vibrio* can become reservoirs of antimicrobial resistance genes [10,11].

The Enterobacteriaceae family comprises Gram-negative bacteria that affect humans, causing urinary tract infections, pneumoniae, gastroenteritis, meningitis, and sepsis [12]. Enterobacteriaceae possess a bacilli shape, are non-spore-forming, have variable motility, and are generally found inside other organisms. Usually, these microorganisms have the ability to mobilise their antimicrobial genetic determinants in plasmids via conjugation, which means that they can be resistant to almost every existing antibiotic [12]. Hospitals throughout the world have been reporting clinical cases of people that contract severe bacterial infections due to the presence of resistant lineages of major infectious agents such as *Escherichia coli* [13] and *Klebsiella pneumoniae* [14].

The abusive and erroneous use of antibiotics by human and veterinary medicine [15,16] promotes a selective pressure for antimicrobial resistant bacteria. The increase of resistant strains is leading to an emerging threat to public health [17,18]. It is estimated that 10 million lives could be lost due to antimicrobial resistant bacteria annually by 2050, if no action is taken [19].

For many years (1950–1970), polymyxin antibiotics were commonly employed in clinical use, until they were gradually withdrawn because of toxicity issues. However, nowadays, the available antibiotics are not enough to cope with the emergence of multidrug-resistance bacteria, resulting in the re-introduction of polymyxins as the last line of defence for clinical treatment [20]. Among polymyxins, the two most used are polymyxin E (colistin) and polymyxin B [21]. Until recently, all the findings about polymyxin-resistant mechanisms were chromosomally mediated [22,23,24]. However, the plasmid-mediated colistin resistance gene *mcr* has already been reported. The first report of *mcr-1* was in *E. coli* and *K. pneumoniae* [25] in China. Since then, the *mcr-1* gene has emerged in strains collected in many parts of the world (e.g., Europe—[26,27,28,29]; Asia—[30,31]; North America—[32]; Africa—[33]). A very large study of bacterial genomes concluded that almost all mobile colistin resistance genes (from *mcr-1* to *mcr-9*) originated from environmental bacteria, mainly from water sources [34]. The origin of *mcr-1* is believed to be from *Moraxella* species [34,35]. As far as the authors know, up to this date, the presence of *mcr-1* has been mostly reported in the Enterobacteriaceae family and only once in a non-Enterobacteriaceae, *Vibrio parahaemolyticus* [36].

Bivalve molluscs such as mussels, clams, or oysters are commercially valuable as a food delicacy. It means that these organisms are regularly monitored for the presence of *Vibrio* spp. and enteric bacteria as indicative of pollution [37]. Furthermore, bivalves are filter-feeders and can filtrate between 20 and 100 L of water per day (species-specific), retaining several microorganisms [38,39].

In this report, environmental bacteria were isolated from two clam species, *Ruditapes decussatus,* and *Ruditapes philippinarum*, after selective isolation for *Vibrio* microorganisms and coliform bacteria. The aim of this study was to evaluate the profile of antimicrobial resistance to three important groups of antimicrobials (polymyxins, carbapenems, and quinolones) in the microbial isolates, and the evaluation of the presence of virulence genes. Three *Vibrio* spp. were found resistant to colistin, with the presence of *mcr-1*, and one of them also presented the virulent *chiA* gene. The phenotypic and phylogenetic analysis strongly support that the three isolates belong to the genus *Vibrio* and represent undescribed species.

## 2. Materials and Methods

### 2.1. Study Area, Sample Collection and Microbial Isolation

**Figure 1:**
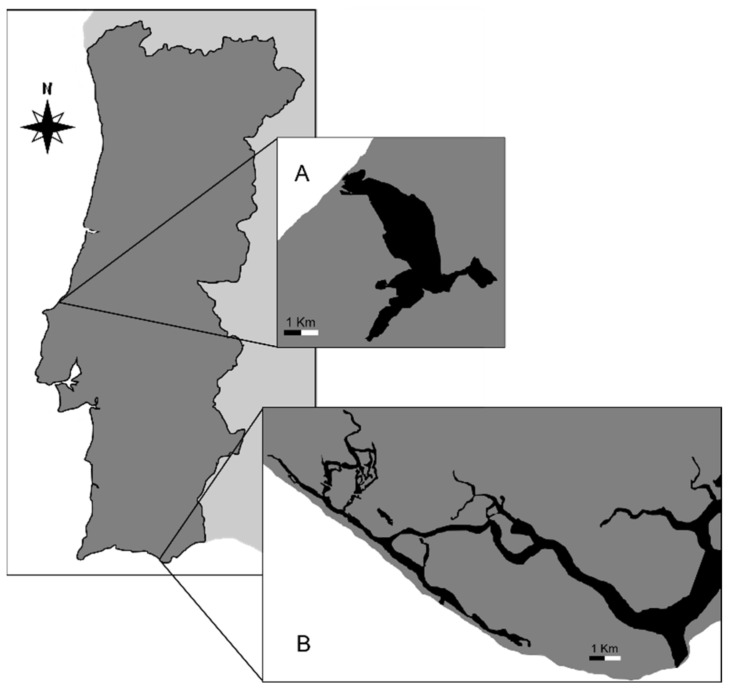
Sampling locations: (**A**) Óbidos Lagoon (39°24′44.1″ N 9°12′54.0″ W) and (**B**) Ria Formosa (37°01′05.4″ N 8°00′11.6″ W).

Individuals of *R. decussatus* and *R. philippinarum* were collected in the Óbidos Lagoon (Caldas da Rainha, Portugal, 39°24′44.1″ N 9°12′54.0″ W, in November of 2019). And other individuals of *R. decussatus* were collected in Ria Formosa (Algarve, Portugal, 37°01′05.4″ N 8°00′11.6″ W, in January of 2020) (Figure 1). The clams were transported, under controlled temperature (10 °C), to the laboratory and immediately processed upon arrival.

Enumeration of Vibrionaceae and coliforms was performed by serial dilutions, of the molluscs edible part and inter-valve liquid, in saline solution (0.85% *w*/*v*) and plating in thiosulfate citrate bile salts sucrose agar (TCBS; VWR, Radnor, PA, USA) and Chromocult^®^ Coliform agar (Merck, Darmstadt, Germany), respectively.

Plates were incubated for 24 h at 28 °C for *Vibrio* spp. and at 37 °C for coliforms. Colony-Forming Units (CFUs) with distinct morphology on TCBS agar were inoculated onto Tryptic Soy Agar (TSA; Biolife, Milano, Italy) supplemented with NaCl (1.5% *w*/*v*), and incubated for 24 h at 28 °C. CFU that had distinct morphology on Chromocult^®^ were inoculated onto Nutrient Agar (NA; VWR, Radnor, PA, USA), and incubated for 24 h at 37 °C. The isolates were conserved at −80 °C in cryovials (VWR, Radnor, PA, USA).

### 2.2. Phenotypic Characterisation of Isolates

#### 2.2.1. Antimicrobial Susceptibility Test

Antimicrobial susceptibility tests were performed by the disk diffusion method, according to the EUCAST guidelines [40] against the antimicrobial: colistin (10 μg), cefotaxime (30 μg), imipenem (10 μg), ciprofloxacin (5 μg) and meropenem (10 μg). The disks were from Thermo Fisher Scientific. Briefly, a microbial suspension in 0.85% saline solution was adjusted to the 0.5 McFarland turbidity standard on a densitometer (VWR, Radnor, PA, USA). For coliforms, the colonies were grown in Mueller-Hinton agar (MHA) plates (Thermo Fisher Scientific, Waltham, MA, USA), at 37 °C for 24 h, and for Vibrionaceae in MHA with 1.5% (*w*/*v*) NaCl at 28 °C for 24 h. The antimicrobial inhibition halos were then measured after this period.

The minimum inhibition concentration (MIC) was determined by the microdilution method, according to the EUCAST guidelines [41], with the addition of 2% (*w*/*v*) NaCl. The plates were incubated at 28 °C for 24 h and the MIC was recorded as the lowest concentration of the colistin that completely inhibited the growth.

#### 2.2.2. Biochemical Characterisation

The Gram staining procedure was performed according to Coico [42] together with the KOH test [43]. Oxidase reaction was made using Cytochrome Oxidase test (bioMérieux, Marcy l’Etoile, France). The biochemical analysis of the three isolates was conducted using API 20NE (bioMérieux, Marcy l’Etoile, France) at 28 °C. The inocula were prepared in saline solution (2% *w*/*v*), adjusted to the 0.5 McFarland turbidity standard. The galleries were filled according to the manufacturer’s instructions. Reads were carried out at 24 h and 48 h. The tests were performed in triplicate.

#### 2.2.3. Lipid Profile

Each isolate was grown in TSA (BD, Sparks, MD, USA) at 30 °C for 24 ± 1 h. The fatty acids of the isolates were extracted and methylated to fatty acid methyl esters (FAMEs) using the Sherlock Instant FAME^TM^ method (MIDI, Inc., Newark, DE, USA). FAMEs were analysed by gas chromatography on a gas chromatograph (GC Agilent Technologies 6890N, Santa Clara, CA, USA), with a flame detector and a 7683 B series injector, using a 25 m Agilent J&W Ultra 2 capillary column. The FAMEs profile of each isolate was determined by the Sherlock^®^ software package, v. 6.2 (MIDI, Inc.).

### 2.3. Genotypic Characterisation of Isolates

#### 2.3.1. DNA Extraction

The DNA from the bacterial isolates was extracted by a simple boiling method. Briefly, the isolates were grown overnight in Tryptic Soy Broth (TSB; Biolife, Milano, Italy) with a final concentration of 2% (*w*/*v*) NaCl, at 28 °C for Vibrionaceae, and at 37 °C for coliforms. Then, 1 mL of each bacteria suspension was centrifuged (10,000× *g*, 1 min) and the supernatant was removed. The pellet was resuspended in 100 µL of sterile Milli Q water and heated at 100 °C for 10 min, followed by centrifugation at 10,000× *g* for 5 min. The supernatant was collected and kept at −20 °C until further use.

#### 2.3.2. Amplification of the 16S rRNA Gene and Housekeeping Genes, Search for Antimicrobial Resistance and Virulence Genes on the Isolates

The search for antimicrobial resistance genes was performed on all isolates (*n* = 60, 43 presumptive *Vibrio* spp. and 17 coliforms) and included three classes of antimicrobials: polymyxins (*mcr-1, mcr-2, mcr-3, mcr-4, mcr-5, mcr-6, mcr-7, mcr-8, mcr-9*), β-lactams (*bla*_IMP_*, bla*_VIM_*, bla*_KPC_*, bla*_NDM_*, bla*_OXA_*, bla*_CTX-M_*, bla*_SHV_ and *bla*_TEM_), and quinolones (*qnrA, qnrB, qnrC, qnrD, qnrS* and *qepA*). The virulence genes searched for were *chiA, vhpA, luxR, flaC, hlyA, tlh, tdh, trh, ctxA, ompU, zot, tcpI, tcpA, sno*/*sto, vvh, vpi, yrpl, ompk* and *vhh.* The 16S rRNA gene, together with four house-keeping genes (*recA, rpoB, groEl, dnaJ*), was amplified and sequenced for phylogenetic analysis in the three *Vibrio* spp. where *mcr-1* was detected. All primers are described in Table S1.

The PCR reactions consisted of NZYTaq II 2x Green Master Mix (Nzytech, Lisboa, Portugal), primers (0.5 μM), and 5 μL of DNA, for a total volume of 50 μL. The PCR was performed in thermal cycle (BioRad, T100, Hercules, CA, USA), and the conditions were the following: 1 cycle of denaturation at 95 °C for 5 min, followed by 35 cycles of denaturation at 95 °C for 30 s, annealing step between 50 °C and 68 °C for 30 s (primer-specific—see Table S1), an elongation step at 72 °C for 40 s, and a final cycle of elongation at 72 °C for 5 min. PCR products were purified using GeneJet PCR Kit (Thermo Fisher Scientific, Waltham, MA, USA), according to the manufacturer’s instructions and sequenced by Sanger sequencing (StabVida, Lisbon, Portugal). The Basic Local Alignment Search Tool (BLAST^®^; https://blast.ncbi.nlm.nih.gov/Blast.cgi, accessed on 10 May 2020) databases from the National Center for Biotechnology Information (NCBI) was used to identify the isolates to the highest taxonomic level possible. The gene sequences obtained for the three isolates, EB07V, NJ21V and NJ22V have been deposited in GenBank under the following accession numbers: (16S rRNA—OL756099; *recA*—OL828804; *rpoB*—OL828807; *groEl*—OL828810; *dnaJ*—OL828813 and *mcr-1*—OM333890) for EB07V; (*16S* rRNA—OL756100; *recA*—OL828805; *rpoB*—OL828808; *groEl*—OL828811; *dnaJ*—OL828814 and *mcr-1*—OM333891) for NJ21V and (*16S* rRNA—OL756101; *recA*—OL828806; *rpoB*—OL828809; *groEl*—OL828812; *dnaJ*—OL828815 *mcr-1*—OM333892) for NJ22V.

### 2.4. Phylogenetic Analysis

All phylogenetic analyses based on partial sequences of 16S rRNA, *recA, rpoB, groEl* and *dnaJ* were performed on Molecular Evolutionary Genetics Analysis (MEGA-X) software [44]. Multilocus Sequence Alignments (MLSA) with partial concatenated sequences of *recA, rpoB, groEl and dnaJ* from the isolates were aligned by ClustalW with full-length reference genes obtained from closely related species belonging to genus *Vibrio* and other genera (*Photobacterium* and *Grimontia*), available on GeneBank from NCBI (accession numbers are available in Table S2). *Photobacterium damselae* 9046-81 and *Grimontia hollisae* FDAARGOS 111 were used as the outgroup. A phylogenetic tree was constructed with the Maximum Likelihood (ML) method [45] and Kimura 2-parameter model. Initial trees for the heuristic search were obtained by applying the Neighbor-Joining method to a matrix of pairwise distances estimated using the Maximum Composite Likelihood (MCL) approach. A discrete Gamma distribution was used to model evolutionary rate differences among sites (5 categories; +G, parameter = 0.8508). The topologies of the phylogenetic tree were determined using bootstrap analyses based on 1000 replicates.

### 2.5. Evaluation of mcr-1 Mobility

#### 2.5.1. Plasmid Extraction and Bacteria Transformation

The *Vibrio* spp. isolates were grown overnight in TSB supplemented with NaCl (1.5% *w*/*v*), at 28 °C. GeneJET Kit (Thermo Fisher Scientific, Waltham, MA, USA) was used for plasmid extraction, following the manufacturer’s instructions. Transformation by heat shock using TOP10 chemically competent *E. coli* (Thermo Fisher Scientific, Invitrogene, Waltham, MA, EUA) was performed. Briefly, the extracted plasmid was added to TOP10 competent *E. coli* and left on ice for 20 min. Then, the mixture was heated for 50 s at 42 °C and placed on ice for 2 min. 500 μL of Luria-Bertani (LB; VWR, Radnor, PA, USA) medium (at room temperature) was added and the cells were incubated at 37 °C for 1 h. The tubes were centrifuged (10,000× *g*, 1 min) and the supernatant was discarded. The pellet was resuspended and inoculated on LB agar supplemented with colistin (PanReac AppliChem, Barcelona, Spain; 2 mg L^−1^). The plates were incubated overnight at 37 °C.

#### 2.5.2. Plasmid Curing

Bacteria plasmid curing involves the loss of plasmids when the growth occurs in the presence of a plasmid curing agent, like ethidium bromide, acridine orange, or sodium dodecyl sulphate in sublethal concentrations [46]. If bacteria lose antimicrobial resistance after curing, it is reasonable to believe that resistance genes are located in the lost plasmid.

The *Vibrio* spp. isolates were grown overnight at 28 °C, in MHA (Thermo Fisher Scientific, Waltham, MA, EUA) supplemented with 2% NaCl (*w*/*v*) and 2 mg L^−1^ colistin. The inoculum was prepared in a 0.85% (*w*/*v*) saline suspension and adjusted to the 0.5 McFarland turbidity standard. Then, sequential dilutions up to the 10^−3^ dilution were made, and ethidium bromide was added in the concentration of 0.025, 0.05, and 0.1 g L^−1^. The microorganisms were incubated at 28 °C (200 rpm) until their growth reached the end of the exponential phase. After that, the isolates were grown on both MHA with and without colistin (2 mg L^−1^), at 28 °C for 24 h. Curing efficiency was determined by comparing the number of growing colonies.

## 3. Results and Discussion

### 3.1. Enumeration of Vibrionaceae and Coliforms Isolates

The CFU per g of molluscs, of *Vibrio* spp. and coliforms were determined for each clam species isolated at different locations. The counts are available in Table 1.

The CFU with distinct morphology on TCBS and on ChromoCult^®^ Coliform agar were isolated, totalising 38 presumptive *Vibrio* spp. and 17 coliforms. Among the 17 coliforms isolated, nine were *E. coli* (blue/violet colonies) and three were *Citrobacter freundii* (red colonies).

Food safety criteria regarding microorganisms are available at the European Commission regulation [47]. There is increased concern regarding food safety in shellfish products. For live bivalve molluscs, *E. coli* has been used as an indicator of hygiene quality. The method used to enumerate *E. coli* is the most probable number (MPN). The range between 230 and 700 MPN per 100 g of bivalve flesh and intra-valvular liquid, is acceptable for direct human consumption according to ISO TS 16649-3:2015 (https://www.iso.org/standard/56824.html, accessed on 12 December 2021). A standard bacterial analysis does not reveal the presence of enteroviruses or microorganisms of the genus *Vibrio*. Thus, there is a need for considering new sanitary quality indicators [48,49].

### 3.2. Antimicrobial Resistance of the Isolates

All 60 isolates (both *Vibrio* spp. and coliforms) were tested for susceptibility/resistance to colistin, cefotaxime, imipenem, ciprofloxacin, and meropenem (Table S3). Almost all isolates showed some degree of sensitivity since a halo was formed. However, three *Vibrio* spp. isolates, EB07V, NJ21V, and NJ22V, showed phenotypic resistance to colistin since an inhibitory halo was not formed (Table S3). The EUCAST does not have Epidemiological Cut off Values (ECOFF) or clinical breakpoint for *Vibrio* spp. against colistin. Still, considering the ECOFF for *Escherichia coli* [50], the isolates (EB07V, NJ21V and NJ22V) had a MIC of >512 mg L^−1^ and can be considered resistant to colistin. EB07V was isolated from *R. decussatus* collected at Óbidos Lagoon, and NJ21V plus NJ22V from *R. philippinarum* collected at Ria Formosa.

The presence of antimicrobial resistance genes in all 60 isolates was determined and from the 23 antimicrobial resistance genes investigated, only the three isolates that showed phenotypic resistance to colistin presented the *mcr-1* gene (Tables S4–S6). The *mcr-1* gene was confirmed by sequencing the three DNA fragments, and compared to those publicly available (BLAST, https://blast.ncbi.nlm.nih.gov/Blast.cgi, accessed on 21 January 2022). The three *mcr-1* sequences shared 100% of identity with plasmid-mediated *mcr-1* gene sequences published (*E. coli*, *Salmonella enterica* and *Klebsiella pneumoniae*).

Considering these results, the presence of virulence genes was assessed on the three isolates. Out of the 19 virulence genes tested, only the *chiA* gene (chitinase A) was found in EB07V. Chitin is the main structural polysaccharide of crustaceans [51], being a valuable carbon and nitrogen source in marine environments [52]. To use chitin, marine bacteria such as *V. vulnificus* [53], *V. harveyi* [54], or *V. anguillarum*, and *V. parahaemolyticus* [55], require a group of enzymes called chitinases and chitin-binding proteins. Chitinases are able to convert chitin into useful biomolecules [51]. The relationship of *V. cholerae* with live copepods (which possess chitin) has already been observed, resulting in an increase in bacteria survivability and culturability [56]. The associations between *Vibrio* spp. with copepods suggest that copepods may be an environmental reservoir for the pathogens and a source of their dissemination [57]. Studies regarding the consumption of copepod nauplii by clams in freshwater, marine, and estuarine environments have been published [58,59]. This grazing can be termed “incidental predation”. However, bivalves can devastate zooplankton populations [60]. Thus, the isolates from this study could have been transmitted through this interaction between copepods and clams.

Since the three *Vibrio* spp. isolates presented the *mcr-1* gene, it was important to ascertain whether the resistance was plasmidic or genomic. Thus, an attempt to transform competent *E. coli* cells and cure the isolates was conducted. In the case of the transformation, no transformed cells were obtained. When attempting to cure the isolates, no differences were observed when the number of growing colonies in medium MHA with and without colistin were compared (Figure S1). These results indicate that the observed resistance to colistin in all three isolates is chromosomally mediated. Lei et al. [36] found the *mcr-1* gene in *V. parahaemolyticus* isolates obtained from shrimp and tested its mobility by conjugation. Their results show that the *mcr-1* gene is plasmid-mediated, and it is allocated in IncX4 plasmid. Indeed, IncX4 plasmids were previously found to be associated with the dissemination of *mcr-1* in enterobacteria and probably were successfully mobilised to *Vibrio* spp. [36,61]. Through in silico analysis in the database (www.ncbi.nlm.nih.gov, accessed on 12 December 2021), it was possible to verify the presence of *mcr-1* in the genome of two *V. parahaemolyticus* (accession number: NNEB01000260.1 and NNHN01000221.1) isolated in China in 2017 and 2019.

Until 2015, colistin resistance was exclusively related to chromosomic mutations in genes involved in lipid A decoration, i.e., *pmrA/B*, *phoP/Q*, *ccrA/B*, *lpxACD*, or *mgrB* genes that resulted in a modification of bacterial membranes by adding sugar to the lipid A moiety [62,63]. The colistin resistance processes can also involve genes encoding phosphoethanolamine transferase (*PET*) and/or glycosyltransferase proteins that are essential for membrane phospholipid biosynthesis [64,65,66]. It was in 2015 that a mobile colistin resistance mechanism was found for the first time: the *mcr-1* variant encoding for a *PET* [25]. Khedher et al. [34] showed that *PET* genes are ubiquitous in bacteria since they were detected in 1047 species, which could not be explained by the abusive overuse of antibiotics for human and veterinary medicine, or agricultural purposes. These findings highlighted a question regarding the role of these enzymes and suggested a defense system in the biosphere against bacteriophages in aquatic environments, but also against cationic antimicrobial peptides secreted by vertebrates (e.g., humans) and invertebrates. The authors also found that bacteria that are intrinsically resistant to colistin, i.e., *Serratia* spp. and *Proteus* spp., also contain MCR variants. *V. vulnificus* and *V. cholerae* have chromosomally mediated colistin resistance [67]. In the case of the biotype EI Tor of *Vibrio cholerae* O1, for example, an *eptA* gene is present in addition to the *almEFG* operon [68,69,70]. Apparently, it is the simultaneous expression of the *eptA* gene [70] and *almEFG* operon [71,72] that trigger the appropriate lipopolysaccharide-lipid A modification, promoting polymyxin resistance [73]. In the case of *V. vulnificus*, it has been reported that the potassium uptake protein TrkA, was responsible for the resistance to polymyxin B [74]. Taking into account that the three isolates did not pass their colistin resistance gene through horizontal transfer models, the results clearly indicate that the isolates contain the *mcr-1* variant in their chromosomes and not in a plasmid.

### 3.3. Biochemical Analysis of the Isolates

The three isolates, EB07V, NJ21V, and NJ22V were positive in the KOH test which indicates the isolates are Gram-negative. *Vibrio* spp. isolates were biochemically characterised, using API 20NE strips (Table 2).

All three isolates were positive for nitrate reduction and cytochrome oxidase activity, like most members of the genus *Vibrio*, with the exception of *V. metschnikovii* and *V. gazogenes* [75]. In addition, the isolates were positive for tryptophane production, esculin, gelatin hydrolysis, and β-galactosidase activity. Conversely, arginine dihydrolase and urease were negative in all isolates. The isolates were able to assimilate glucose, mannitol, *N*-acetyl-glucosamine, maltose, potassium gluconate, and malate. However, none of the isolates could assimilate arabinose, and capric and phenylacetic acids. Mannose was only assimilated by EB07V and NJ21V. EB07V and NJ22V were not capable of assimilating adipic acid and, in the case of NJ21V, the results were not conclusive. Only EB07V was able to ferment glucose and assimilate trisodium citrate. It is known that *Vibrio* strains have the ability to ferment glucose anaerobically [9,76]. However, NJ21V and NJ22V were unable of doing so. Nevertheless, NJ21V and NJ22V were able to grow on TCBS medium, which has an alkaline pH of 8.6, bile salt, and NaCl which suppress the growth of most interfering bacteria. Thus, due to the selectivity of the medium and subsequent genetic analysis, the isolates NJ21V and NJ22V were considered as *Vibrio* spp.

The lipid profile of microorganisms may be used for their identification [77,78]. The most prevalent fatty acids (Table 3) in the isolates EB07V, NJ21V and NJ22V were 16:0, 18:3 ω6c, summed feature 3 (16:1 ω6c/16:1 ω7c), and summed feature 8 (18:1 ω6c/18:1 ω7c). When compared to other studies that used the same system to analyse the fatty acid profiles of *Vibrio* spp. grown under the same growth conditions, similar profiles were obtained. *V. alginolyticus*, *V. fischeri*, *V. harveyi*, and *V. natriegens* present as major fatty acids 16:0 (13–36%), 18:1 ω7c (15–22%), and summed feature 3 (34–36%) [79]. The cellular fatty acid composition of *V. inhibens* sp. nov. contains similarly mainly 16:0 (14–30%), 18:1 ω7c (8–24%), and summed feature 3 (35–43%) [80]. It has been proposed that *V. inhibens* is a later heterotypic synonym of *V. jasicida* [81]. The most curious fatty acid in the three isolates of the present study is the polyunsaturated 18:3 w6c (Table 3). Nevertheless, the production of polyunsaturated fatty acids has been described in marine and psychrophilic *Vibrio* spp. [82,83].

### 3.4. Phylogenetic Analysis

After comparing the 16S rRNA sequences of the three isolates to those publicly available (BLAST, https://blast.ncbi.nlm.nih.gov/Blast.cgi, accessed on 21 December 2021), the isolates were identified as belonging to the *Vibrio* genus, but it was not possible to identify them to the species level. The isolate EB7V shared 99.47% of similarity with *V. hangzhouensis* and *V. mediterranei*; NJ21V shared 98.68% of similarity with several *Vibrio* spp. (e.g., *V. xuii, V. parahaemolyticus*, *V. galatheae*); and NJ22V shared 98.71% of similarity with some *Vibrio* spp. (e.g., *V. xuii*, *V. parahaemolyticus*, *V. scophthalmi*). The genus *Vibrio* contains a large number of closely related species sharing a high level of sequence similarity with just 0.1 or 0.2% difference in the nucleotide sequence of 16S rRNA [84]. Based on that, the use of other genes (e.g., *rpoA*, *recA*, *pyrH*, and *atpA*) to discriminate closely related species has been proposed [85,86]. Four other house-keeping genes were chosen, *recA*, *rpoB*, *groEL*, and *dnaJ* that were sequenced and aligned. The gene *groEL*, for example, was reported to be extremely conserved in nature [87] and was already used to identify *Vibrio* isolates [88].

**Figure 2:**
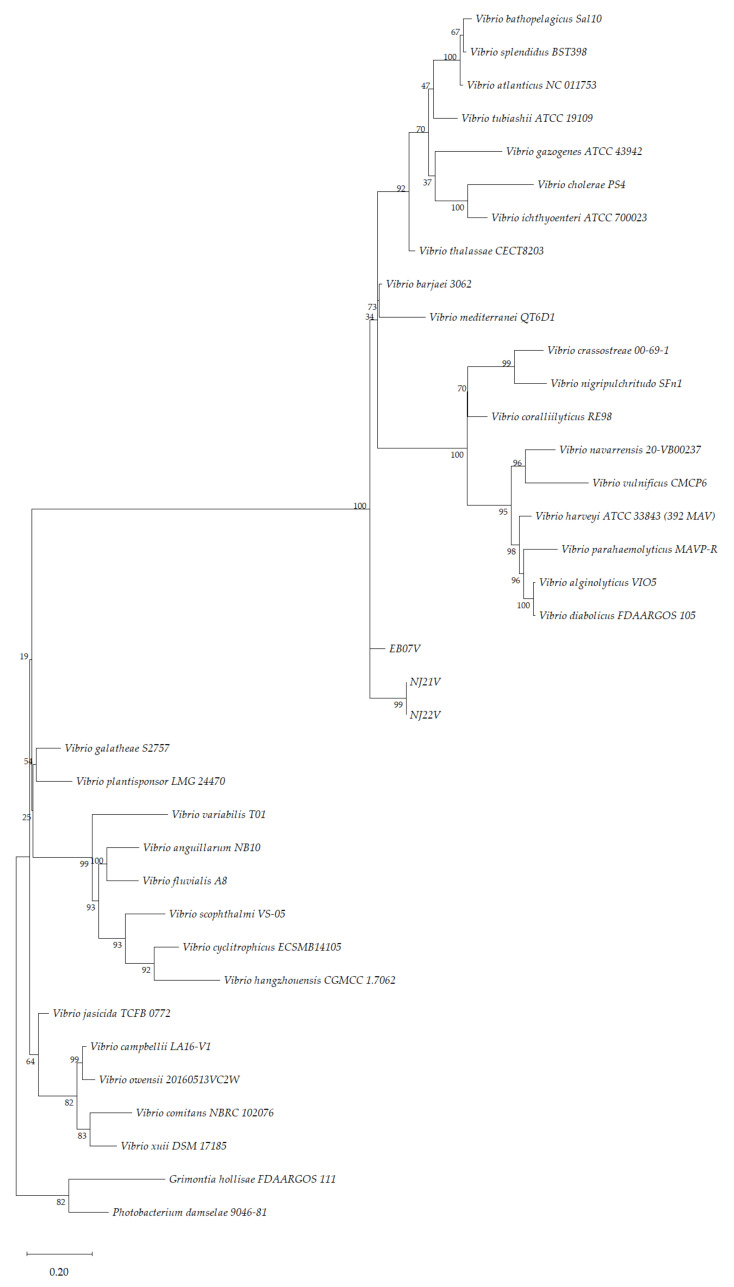
Phylogenetic construction based on the concatenation of partial sequences of four house-keeping genes (*recA*, *rpoB*, *groEL*, and *dnaJ*) by the ML method algorithm and Kimura 2-parameter model [94]. The tree with the highest log likelihood (−140419,60) is shown. The percentage of trees in which the associated taxa clustered together is shown next to the branches. Initial trees for the heuristic search were obtained by applying the Neighbor-Joining method to a matrix of pairwise distances estimated using the Maximum Composite Likelihood (MCL) approach. A discrete Gamma distribution was used to model evolutionary rate differences among sites (5 categories; +G, parameter = 0.8284)). The tree was drawn to scale, with branch lengths measured in the number of substitutions per site, 0.20. There were a total of 8130 positions in the final dataset. *Photobacterium damselae* 9046-81 and *Grimontia hollisae* FDAARGOS 111 were used as the outgroup.

The phylogenetic tree for concatenated sequences for the house-keeping genes was constructed using the ML method (Figure 2). The MLSA is based on partial sequences of the four house-keeping genes for 32 *Vibrio* spp., *Photobacterium damselae* 9046-81 and *Grimontia hollisae* FDAARGOS 111 (used as outgroup) and the three isolates. MLSA has been proposed as a useful technique for the identification of *Vibrio* isolates and for studying the phylogeny in this genus [85,89]. Moreover, this technique has been utilised for the identification of some new *Vibrio* species, such as *V. gigantis* and *V. crassostreae* [90,91] and, more recently for *V. barjaei* and *V. nitrifigilis* [92,93].

However, the three isolates clustered in the genus *Vibrio* but do not cluster with any *Vibrio* clade, which suggests that the three isolates may represent new undescribed species of the genus *Vibrio*. In the case of NJ21V and NJ22V, it appears that they belong to the same species, although being different strains. Further studies using these isolates are needed to prove this statement.

## 4. Conclusions

In this study, the presence of the mobilisable colistin resistance gene *mcr-1* in three *Vibrio* spp. (EB07V, NJ21V, and NJ22V), isolated in microbial communities from clams, highlights a potential threat to public health. Clams are an appreciated food delicacy and colistin is one of the last line of defence antibiotics used for clinical treatment [20]. One of the three *mcr-1* positives isolates, EB07V, also carried the gene *chiA,* responsible for the invasion of tissues of chitin-containing organisms, which may increase its pathogenic potential. The location of the *mcr-1* resistance gene appears to be chromosomal which indicates a low potential for mobilisation. Biochemical analysis and MLSA on the basis of four protein-coding gene sequences (*recA, rpoB*, *groEL* and *dnaJ*) grouped the isolates into the genus *Vibrio* but distinguished them as different from other species of *Vibrio,* suggesting we might be in the presence of new species. To corroborate this statement further investigation is required. However, the fact that the microbial communities in the environment possess the colistin mobilise resistant determinant *mcr-1,* highlights the importance of this study.

## Figures and Tables

**Table 1:** The CFU per g of molluscs, of *Vibrio* spp. and coliforms for each clam species isolated at different locations.

Isolates	Óbidos Lagoon		Ria Formosa
	*R. decussatus* (CFU g^−1^)	*R. phillipinarum* (CFU g^−1^)	*R. decussatus* (CFU g^−1^)
Vibrionaceae	1.1 × 10^4^	4.5 × 10^3^	3.8 × 10^3^
Coliforms	8.7 × 10^3^	3.5 × 10^3^	5.0 × 10^2^

**Table 2:** Biochemical characterisation of the three isolates that show colistin resistance. A “+” indicates a positive reaction, whereas a “−“ denotes a negative reaction. NC—not conclusive.

Test	EB07V	NJ21V	NJ22V
Nitrate reduction	+	+	+
Tryptophane production	+	+	+
Glucose fermentation	+	−	−
Arginine dihydrolase	−	−	−
Urease	−	−	−
Esculin (β-glucosidase activity)	+	+	+
Gelatin (protease activity)	+	+	+
*Para*-NitroPhenyl-β-D-Galactopyranosidase (β-galactosidase activity)	+	+	+
Glucose assimilation	+	+	+
Arabinose assimilation	−	−	−
Mannose assimilation	+	+	−
Mannitol assimilation	+	+	+
*N*-Acetyl-Glucosamine assimilation	+	+	+
Maltose assimilation	+	+	+
Potassium gluconate assimilation	+	+	+
Capric acid assimilation	−	−	−
Adipic acid assimilation	−	NC	−
Malate assimilation	+	+	+
Trisodium citrate assimilation	+	−	−
Phenylacetic acid assimilation	−	−	−
Cytochrome oxidase	+	+	+

**Table 3:** Fatty acid profiles, in % of total fatty acids, of the three isolates.

Fatty Acid	EB07V	NJ21V	NJ22V
12:0	3.72	8.11	7.22
14:0 *iso*	1.13		
14:0	5.29	3.12	2.70
15:0 *iso*	0.96		
16:0 *iso*	1.28	0.82	0.88
16:0	15.85	25.05	26.87
17:0 *iso*	1.76		
18:3 w6c	14.24	20.78	12.17
18:0	3.60	2.88	2.43
Summed Feature 3	30.46	24.53	26.59
Summed Feature 8	21.15	12.68	18.10

## Supplementary Materials

The following are available online at https://www.mdpi.com/article/10.3390/microorganisms10020394/s1, Table S1: Primers sequences used to amplify house-keeping, antimicrobial resistances and virulence genes. Table S2: Accession numbers from NCBI of the species used to performed MLSA on MEGA X software. Table S3: Antimicrobial susceptibility test using colistin, imipenem, cefotaxime, meropenem and ciprofloxacin disks. The halo measurements displayed in mm. Table S4: Antimicrobial resistances genes search on all the isolates. The first class of antibiotics searched was polymyxin (*mcr-1, mcr-2, mcr-3, mcr-4, mcr-5, mcr-6, mcr-7, mcr-8* and *mcr-9*). – indicates non detected gene and + indicates detected gene. Table S5: Antimicrobial resistances genes search on all the isolates. The second class was β-lactams (*bla*_IMP_, *bla*_VIM_, *bla*_KPC_, *bla*_NDM_, *bla*_OXA_, *bla*_CTX-M_, *bla*_SHV_ and *bla*_TEM_). – indicates non detected gene and + indicates detected gene. Table S6: Antimicrobial resistances genes search on all the isolates. The third antimicrobial class searched was quinolone (*qnrA, qnrB, qnrC, qnrD, qnrS* and *qepA*). – indicates non detected gene and + indicates detected gene. Figure S1: Plasmid curing of the isolates EB07V, NJ21V and NJ22V. The number of CFU was determined in MHA plates with and without colistin. References [95,96,97,98,99,100,101,102,103,104,105,106,107,108,109,110,111,112,113,114,115,116,117,118] have been cited in the Supplementary materials.
