# Supplementary material for: Detection of mcr-1 Gene in Undefined Vibrio Species Isolated from Clams
Source: Microorganisms. 2022 Feb 8;10(2):394. doi: 10.3390/microorganisms10020394 (PMC8876837; doi:10.3390/microorganisms10020394)
Supplement: Supplementary file 1 [file microorganisms-10-00394-s001.zip › microorganisms-1546984-supplementary.pdf]

Detection of *mcr-1* gene in undefined *Vibrio* species isolated from clams

Christian Valdez [1†], Cátia Costa [1†], Marco Simões [1], Carla C.C.R. de Carvalho[2], Teresa Baptista[1] and Maria Jorge Campos[1*]

[1] MARE-Marine and Environmental Sciences Centre, ESTM, Polytechnic of Leiria, 2520-630 Peniche, Portugal; catia.g.costa@ipleiria.pt (C. C.); 4180268@my.ipleiria.pt (C. V.); marco.a.simoes@ipleiria.pt (M. S.); teresa.baptista@ipleiria.pt (T. B.)

[2] iBB-Institute for Bioengineering and Biosciences, Department of Bioengineering, Associate Laboratory i4HB—Institute for Health and Bioeconomy, Instituto Superior Técnico, Universidade de Lisboa, , Av. Rovisco Pais, 1049-001 Lisboa, Portugal; ccarvalho@tecnico.ulisboa.pt (C. C.)

* Correspondence: mcampos@ipleiria.pt (M. J. C.)

† These authors contributed equally to the development of this study

Supplementary Materials

Table S1. The five house-keeping genes (*recA, rpoB, groEl, 16S* and *dnaJ*) and respective primers that were used to identify the isolates. The primers that were used to test antimicrobial resistances genes presence belongs to three classes. The first class of antibiotics searched was polymyxins (*mcr-1, mcr-2, mcr-3, mcr-4, mcr-5, mcr-6, mcr-7, mcr-8* and *mcr-9*). The second class was β- lactams (*bla*IMP, *bla*VIM, *bla*KPC, *bla*NDM, *bla*OXA, *bla*CTX-M, *bla*SHV and *bla*TEM). The third antimicrobial class searched was quinolone (*qnrA, qnrB, qnrC, qnrD, qnrS* and *qepA*). The virulence genes searched in the three isolates colistin resistant were *chiA, vhpA, luxR, flaC, hlyA, tlh, tdh, trh, ctxA, ompU, zot, tcpI, tcpA, sno/sto, vvh, vpi, yrpl, ompk* and *vhh*. .

Genes	FW	REV	TA (°C)	Reference
recA	5′-GTCTACCAATGGGTCGTATC-3′	5′-GCCATTGTAGCTGTACCAAG-3′	60	[95]
rpoB	5′-GAGTCTTCGAAGTTGTAACC-3′	5′-GGCGAAATGGCWGAGAACCA-3′	51.5	[96]
groEl	5′-TCCARAACATGGGGCGCACAA-3′	5′-ACGTTTTGYTCTTCGTTGTCRC-3′	68	[97]
***16S* rRNA**	5′-AGAGTTTGATCCTGGCTCAG-3′	5′-GGTTACCTTGTTACGACTT-3′	56	[98]
dnaJ	5′- GATYTRCGHTAYAACATGGA-3′	5′- TTCACRCCRTYDAAGAARC-3′	50	[99]
mcr-1	5′-AGTCCGTTTGTTCTTGTGGC-3′	5′-AGATCCTTGGTCTCGGCTTG-3′	60	[100]
mcr-2	5′- CAAGTGTGTTGGTCGCAGTT-3′	5′-TCTAGCCCGACAAGCATACC-3′	58	[100]
mcr-3	5′- AAATAAAAATTGTTCCGCTTATG-3′	5′-AATGGAGATCCCCGTTTTT-3′	58	[100]
mcr-4	5′-TCACTTTCATCACTGCGTTG-3′	5′-TTGGTCCATGACTACCAATG-3′	58	[100]
mcr-5	5′-ATGCGGTTGTCTGCATTTATC-3′	5′-TCATTGTGGTTGTCCTTTTCTG-3′	58	[100]
mcr-6	5′-GTCCGGTCAATCCCTATCTGT-3′	5′-ATCACGGGATTGACATAGCTAC-3′	58	[100]
mcr-7	5′-TGCTCAAGCCCTTCTTTTCGT-3′	5′-TTCATCTGCGCCACCTCGT-3′	55	[101]
mcr-8	5′-AACCGCCAGAGCACAGAATT-3′	5′-TTCCCCCAGCGATTCTCCAT-3′	60	[101]
mcr-9	5′-CGGTACCGCTACCGCAATAT-3′	5′-ATAACAGCGAGACACCGGTT-3′	58	[101]
***bla*IMP**	5′-GGAATAGAGTGGCTTAAYTCTC-3′	5′-GGTTTAAYAAAACAACCACC-3′	50	[102]
***bla*VIM**	5′-GATGGTGTTTGGTCGCATA-3′	5′-CGAATGCGCAGCACCAG-3′	55	[102]

***bla*$_{KPC}$**	5′-CGTCTAGTTCTGCTGTCTTG-3′	5′-CTTGTCATCCTTGTTAGGCG-3′	52	[102]
***bla*$_{NDM}$**	5′-GGTTTGGCGATCTGGTTTTC-3′	5′-CGGAATGGCTCATCACGATC-3′	52	[102]
***bla*$_{OXA}$**	5′-GCGTGGTTAAGGATGAACAC-3′	5′-CATCAAGTTCAACCCAACCG-3′	52	[103]
***bla*$_{CTX-M}$**	5′-ATGTGCAGYACCACTAARGTKATGGC-3′	5′-TGGGTRAARTARGTSAC-CAGAAYCAGCGG-3′	60	[102]
***bla*$_{SHV}$**	5′-ATGCGTTATATTCGCCTGTG-3′	5′-TGCTTTGTTATTCGGGCCAA-3′	55	[102]
***bla*$_{TEM}$**	5′-TCGCCGCATACACTATTCTCAGAATGA-3′	5′-ACGCTCACCGGCTCCAGATTTAT-3′	50	[102]
qnrA	5′-AGAGGATTTCTCACGCCAGG-3′	5′-TGCCAGGCACAGATCTTGAC-3′	54	[104]
qnrB	5′-GGMATHGAAATTCGCCACTG-3′	5′-TTTGCYGYYCGCCAGTCGAA-3′	54	[104]
qnrC	5′-GGGTTGTACATTTATTGAATC-3′	5′-TCCACTTTACGAGGTTCT-3′	50	[105]
qnrD	5′-CGAGATCAATTTACGGGGAATA-3′	5′-AACAAGCTGAAGCGCCTG-3′	50	[105]
qnrS	5′-GCAAGTTCATTGAACAGGGT-3′	5′-TCTAAACCGTCGAGTTCGGCG-3′	54	[104]
qepA	5′-AACTGCTTGAGCCCGTAGAT-3′	5′-GTCTACGCCATGGACCTCAC-3′	50	[106]
chiA	5′-GGAAGATGGCGTGATTGACT-3′	5′-GGCATCAATTTCCCAAGAGA-3′	50	[107]
vhpA	5′- CTGAACGACGCCCATTATTT-3′	5′-CGCTGACACATCAAGGCTAA-3′	50	[107]
luxR	5′- ATGGACTCAATTGCAAAGAG-3′	5′-TTAGTGATGTTCACGGTTGT-3′	50	[107]
flaC	5′-AAATCATTCCAAATCGGTGC-3′	5′-TCTTTGATTCGGCTCTTA-3′	58	[108]
hlyA	5′-GGCAAACAGCGAAACAAATACC-3′	5′-CTCAGCGGGCTAATACGGTTTA-3′	60	[109]
tlh	5′-AAAGCGGATTATGCAGAAGCACTG-3′	5′-GCTACTTTCTAGCATTTTCTCTGC-3′	54	[110]
tdh	5′- GTAAAGGTCTCTGACTTTTGGAC-3′	5′- TGGAATAGAACCTTCATCTTCACC-3′	58	[110]
trh	5′- TTGGCTTCGATATTTTCAGTATCT-3′	5′-CATAACAAACATATGCCCATTTCCG-3′	58	[110]
ctxA	5′-CGGGCAGATTCTAGACCCTCCTG-3′	5′-CGATGATCTTGGAGCATT CCCAC-3′	62	[111]
ompU	5′-ACGCTGACGGAATCA ACCAAAG-3′	5′-GCGGAAGTTTGGCTTGAA GTAG-3′	61	[111]
zot	5′- TCGCTTAACGATGGC GCGTTTT-3′	5′-AACCCCGTTTCACTTCTA CCCA-3′	61	[112]
tcpI	5′-TAGCCTTAGTTCTCA GCAGGCA-3′	5′-GGCAATAGTGTCGAGCTC GTTA-3′	60	[112]
tcpA	5′-CCTTCGATCCCCTAA GCAATAC-3′	5′-AGGGTTAGCAACGATGCG TAAG-3′	60	[112]
sno/sto	5′- TCGCATTTAGCCAAA CAGTAGAAA-3′	5′-GCTGGATTGCAACATATT TCGC-3′	55	[112]
vvh	5′-GCTATTTCACCGCCGCTCAC-3′	5′--CCGCAGAGCCGTAAACCGAA-3′	55	[113]
vpi	5′-GCAATTTAGGGGCGCGACGT-3′	5′-CCGCTCTTTCTTGATCTGGTAG-3′	52	[114]
yrpl	5′-TATTCAACTGAAAGTGTA-3′	5′-ATAGCTCATAATACTGA-3′	60	[115]
ompk	5′-GGCGGTCGCTCTGGTATT-3′	5′-TTGCCATCGTAAGTGCTGTA-3′	55	[116]
vhh	5′-ATCATGAATAAAACTATTACGTTACT-3′	5′-GAAAGGATGGTTTGACAAT-3′	55	[117]
toxR	5′-GATTAGGAAGCAACGAAAG-3′	5′-GCAATCACTTCCACTGGTAAC-3′	54	[118]

Table S2. Accession numbers from NCBI of the species used to performed MLSA on MEGA X software.

Species strain	Accession number
Vibrio alginolyticus VIO5	AP022861.1
Vibrio anguillarum NB10	LK021130.1
Vibrio atlanticus NC_011753	NC_011753.2
Vibrio barjaei 3062	NR_152641.1
Vibrio bathopelagicus Sal10	NZ_CP062500.1
Vibrio campbellii LA16-V1	NZ_CP021145.1
Vibrio comitans NBRC 102076	NZ_BJLH01000038.1
Vibrio coralliilyticus RE98	CP009617.1
Vibrio crassostreae 00-69-1	NZ_VTXY01000034.1
Vibrio cholerae PS4	NZ_CP077197.1
Vibrio cyclitrophicus ECSMB14105	NZ_CP039700.1
Vibrio diabolicus FDAARGOS_105	NZ_CP014036.1
Vibrio fluvialis A8	NZ_CP053664.1
Vibrio galatheae S2757	JXXV01000001.1
Vibrio gazogenes ATCC 43942	NZ_CP018835.1
Vibrio hangzhouensis CGMCC 1.7062	NZ_FNVG01000060.1
Vibrio harveyi ATCC 33843 (392 [MAV])	NZ_CP009467.1
Vibrio ichthyoenteri ATCC 700023	AFWF01000003.1
Vibrio jasicida TCFB 0772	NZ_BAOG01000278.1
Vibrio mediterranei QT6D1	NZ_CP018308.1
Vibrio mediterranei 117-T6	NZ_CP033579.1
Vibrio mediterranei Vic-OC-097	NZ_JABXGK010000008.1
Vibrio mediterranei V7A	NZ_PYVE01000089.1
Vibrio navarrensis 20-VB00237	CP065217.1
Vibrio nigripulchritudo SFn1	FO203527.1
Vibrio owensii 20160513VC2W	CP030798.1
Vibrio parahaemolyticus MAVP-R	CP022552.2
Vibrio plantisponsor LMG 24470	NZ_JABEQC000000000.1
Vibrio scophthalmi VS-05	NZ_CP016414.1
Vibrio splendidus BST398	NZ_CP031055.1
Vibrio thalassae CECT 8203	NR_134228.1
Vibrio tubiashii ATCC 19109	NZ_CP009354.1
Vibrio variabilis T01	KP329555.1
Vibrio vulnificus CMCP6	NC_004459.3
Vibrio xuii DSM 17185	MH315871.1
Grimontia hollisae FDAARGOS_111	NZ_CP014056.2
Photobacterium damselae 9046-81	NZ_CP046752.1

Table S3. Antimicrobial susceptibility test using colistin, imipenem, cefotaxime, meropenem and ciprofloxacin disks. The halo measurements displayed in mm.

Isolate	Colistin (mm)	Imipenem (mm)	Cefotaxime (mm)	Meropenem (mm)	Ciprofloxacin (mm)
**NB04V**	13	32	37	30	27
**NB08V**	20	35	37	39	32
**NB09V**	22	25	32	32	34
**NB10V**	35	40	37	36	35
**NB11V**	22	32	35	36	26
**NB12V**	25	33	30	35	35
**NB13V**	40	39	40	37	36
**NB14V**	39	40	36	38	36
**NB15V**	18	30	36	34	27
**NB16B**	22	30	36	33	28
**NB17V**	22	31	35	34	29
**NB18V**	22	32	37	35	27
**NB19V**	13	30	34	32	28
**NB20V**	14	34	37	40	29
**NB21V**	20	34	40	36	30
**NJ01V**	19	30	36	33	28
**NJ02V**	18	34	38	32	30
**NJ04V**	19	35	40	33	29
**NJ06V**	13	32	37	32	26
**NJ07V**	15	27	35	32	27
**NJ10V**	40	37	36	40	34
**NJ12V**	20	34	40	35	30
**NJ13V**	20	34	34	35	28
**NJ14V**	22	30	36	35	35
**NJ15V**	20	30	34	36	27
**NJ18V**	10	25	30	36	29
**NJ21V**	0	35	35	32	35
**NJ22V**	0	34	37	33	32
**NB01E**	14	35	25	30	40
**NB02E**	13	32	17	26	30
**NB03E**	12	32	35	35	34
**NB04E**	12	30	32	33	33
**NB05E**	12	29	33	33	37
**NJ01E**	14	32	20	22	32
**NJ02E**	12	29	32	32	35
**NJ03E**	13	32	35	35	33
**NJ04E**	12	30	33	30	32
**NJ05E**	13	29	32	32	30
**NJ08E**	13	30	32	33	27
**NJ09E**	12	27	32	33	33
**NJ10E**	13	30	36	36	35
**NJ11E**	15	32	20	27	35
**NJ12E**	13	31	33	34	30
**EB01V**	20	34	36	36	32
**EB02V**	15	34	40	36	27
**EB03V**	15	35	40	32	28
**EB04V**	15	34	38	35	26
**EB05V**	16	35	32	30	37
**EB06V**	21	30	34	32	36
**EB07V**	0	28	34	30	36
**EB08V**	16	28	32	34	25
**EB09V**	20	40	36	36	35

**EB10V**	16	32	35	33	24
**EB11V**	26	38	40	37	32
**EB12V**	16	33	35	35	29
**EB13V**	20	39	40	40	32
**EB14V**	15	35	40	38	25
**EB15V**	15	30	36	35	27
**EB01E**	10	28	33	35	36
**EB02E**	12	35	32	37	37

Table S4. Antimicrobial resistances genes search on all the isolates. The first class of antibiotics searched was polymyxin (*mcr-1, mcr-2, mcr-3, mcr-4, mcr-5, mcr-6, mcr-7, mcr-8* and *mcr-9*). – indicates non detected gene and + indicates detected gene.

Isolate	*mcr-1*	*mcr-2*	*mcr-3*	*mcr-4*	*mcr-5*	*mcr-6*	*mcr-7*	*mcr-8*	*mcr-9*
**NB04V**	-	-	-	-	-	-	-	-	-
**NB08V**	-	-	-	-	-	-	-	-	-
**NB09V**	-	-	-	-	-	-	-	-	-
**NB10V**	-	-	-	-	-	-	-	-	-
**NB11V**	-	-	-	-	-	-	-	-	-
**NB12V**	-	-	-	-	-	-	-	-	-
**NB13V**	-	-	-	-	-	-	-	-	-
**NB14V**	-	-	-	-	-	-	-	-	-
**NB15V**	-	-	-	-	-	-	-	-	-
**NB16B**	-	-	-	-	-	-	-	-	-
**NB17V**	-	-	-	-	-	-	-	-	-
**NB18V**	-	-	-	-	-	-	-	-	-
**NB19V**	-	-	-	-	-	-	-	-	-
**NB20V**	-	-	-	-	-	-	-	-	-
**NB21V**	-	-	-	-	-	-	-	-	-
**NJ01V**	-	-	-	-	-	-	-	-	-
**NJ02V**	-	-	-	-	-	-	-	-	-
**NJ04V**	-	-	-	-	-	-	-	-	-
**NJ06V**	-	-	-	-	-	-	-	-	-
**NJ07V**	-	-	-	-	-	-	-	-	-
**NJ10V**	-	-	-	-	-	-	-	-	-
**NJ12V**	-	-	-	-	-	-	-	-	-
**NJ13V**	-	-	-	-	-	-	-	-	-
**NJ14V**	-	-	-	-	-	-	-	-	-
**NJ15V**	-	-	-	-	-	-	-	-	-
**NJ18V**	-	-	-	-	-	-	-	-	-
**NJ21V**	+	-	-	-	-	-	-	-	-
**NJ22V**	+	-	-	-	-	-	-	-	-
**NB01E**	-	-	-	-	-	-	-	-	-
**NB02E**	-	-	-	-	-	-	-	-	-
**NB03E**	-	-	-	-	-	-	-	-	-
**NB04E**	-	-	-	-	-	-	-	-	-
**NB05E**	-	-	-	-	-	-	-	-	-
**NJ01E**	-	-	-	-	-	-	-	-	-
**NJ02E**	-	-	-	-	-	-	-	-	-
**NJ03E**	-	-	-	-	-	-	-	-	-
**NJ04E**	-	-	-	-	-	-	-	-	-
**NJ05E**	-	-	-	-	-	-	-	-	-
**NJ08E**	-	-	-	-	-	-	-	-	-
**NJ09E**	-	-	-	-	-	-	-	-	-
**NJ10E**	-	-	-	-	-	-	-	-	-
**NJ11E**	-	-	-	-	-	-	-	-	-

**NJ12E**	-	-	-	-	-	-	-	-	-
**EB01V**	-	-	-	-	-	-	-	-	-
**EB02V**	-	-	-	-	-	-	-	-	-
**EB03V**	-	-	-	-	-	-	-	-	-
**EB04V**	-	-	-	-	-	-	-	-	-
**EB05V**	-	-	-	-	-	-	-	-	-
**EB06V**	-	-	-	-	-	-	-	-	-
**EB07V**	+	-	-	-	-	-	-	-	-
**EB08V**	-	-	-	-	-	-	-	-	-
**EB09V**	-	-	-	-	-	-	-	-	-
**EB10V**	-	-	-	-	-	-	-	-	-
**EB11V**	-	-	-	-	-	-	-	-	-
**EB12V**	-	-	-	-	-	-	-	-	-
**EB13V**	-	-	-	-	-	-	-	-	-
**EB14V**	-	-	-	-	-	-	-	-	-
**EB15V**	-	-	-	-	-	-	-	-	-
**EB01E**	-	-	-	-	-	-	-	-	-
**EB02E**	-	-	-	-	-	-	-	-	-

Table S5. Antimicrobial resistances genes search on all the isolates. The second class was β- lactams (*bla*IMP, *bla*VIM, *bla*KPC, *bla*NDM, *bla*OXA, *bla*CTX-M, *bla*SHV and *bla*TEM). – indicates non detected gene and + indicates detected gene.

Isolate	***bla*IMP**	*bla*VIM	*bla*KPC	*bla*NDM	*bla*OXA	*bla*CTX-M	*bla*SHV	*bla*TEM
**NB04V**	-	-	-	-	-	-	-	-
**NB08V**	-	-	-	-	-	-	-	-
**NB09V**	-	-	-	-	-	-	-	-
**NB10V**	-	-	-	-	-	-	-	-
**NB11V**	-	-	-	-	-	-	-	-
**NB12V**	-	-	-	-	-	-	-	-
**NB13V**	-	-	-	-	-	-	-	-
**NB14V**	-	-	-	-	-	-	-	-
**NB15V**	-	-	-	-	-	-	-	-
**NB16B**	-	-	-	-	-	-	-	-
**NB17V**	-	-	-	-	-	-	-	-
**NB18V**	-	-	-	-	-	-	-	-
**NB19V**	-	-	-	-	-	-	-	-
**NB20V**	-	-	-	-	-	-	-	-
**NB21V**	-	-	-	-	-	-	-	-
**NJ01V**	-	-	-	-	-	-	-	-
**NJ02V**	-	-	-	-	-	-	-	-
**NJ04V**	-	-	-	-	-	-	-	-
**NJ06V**	-	-	-	-	-	-	-	-
**NJ07V**	-	-	-	-	-	-	-	-
**NJ10V**	-	-	-	-	-	-	-	-
**NJ12V**	-	-	-	-	-	-	-	-
**NJ13V**	-	-	-	-	-	-	-	-
**NJ14V**	-	-	-	-	-	-	-	-
**NJ15V**	-	-	-	-	-	-	-	-
**NJ18V**	-	-	-	-	-	-	-	-
**NJ21V**	-	-	-	-	-	-	-	-
**NJ22V**	-	-	-	-	-	-	-	-
**NB01E**	-	-	-	-	-	-	-	-
**NB02E**	-	-	-	-	-	-	-	-
**NB03E**	-	-	-	-	-	-	-	-
**NB04E**	-	-	-	-	-	-	-	-

**NB05E**	-	-	-	-	-	-	-	-
**NJ01E**	-	-	-	-	-	-	-	-
**NJ02E**	-	-	-	-	-	-	-	-
**NJ03E**	-	-	-	-	-	-	-	-
**NJ04E**	-	-	-	-	-	-	-	-
**NJ05E**	-	-	-	-	-	-	-	-
**NJ08E**	-	-	-	-	-	-	-	-
**NJ09E**	-	-	-	-	-	-	-	-
**NJ10E**	-	-	-	-	-	-	-	-
**NJ11E**	-	-	-	-	-	-	-	-
**NJ12E**	-	-	-	-	-	-	-	-
**EB01V**	-	-	-	-	-	-	-	-
**EB02V**	-	-	-	-	-	-	-	-
**EB03V**	-	-	-	-	-	-	-	-
**EB04V**	-	-	-	-	-	-	-	-
**EB05V**	-	-	-	-	-	-	-	-
**EB06V**	-	-	-	-	-	-	-	-
**EB07V**	-	-	-	-	-	-	-	-
**EB08V**	-	-	-	-	-	-	-	-
**EB09V**	-	-	-	-	-	-	-	-
**EB10V**	-	-	-	-	-	-	-	-
**EB11V**	-	-	-	-	-	-	-	-
**EB12V**	-	-	-	-	-	-	-	-
**EB13V**	-	-	-	-	-	-	-	-
**EB14V**	-	-	-	-	-	-	-	-
**EB15V**	-	-	-	-	-	-	-	-
**EB01E**	-	-	-	-	-	-	-	-
**EB02E**	-	-	-	-	-	-	-	-

Table S6. Antimicrobial resistances genes search on all the isolates. The third antimicrobial class searched was quinolone (*qnrA, qnrB, qnrC, qnrD, qnrS* and *qepA*). – indicates non detected gene and + indicates detected gene.

Isolate	*qnrA*	*qnrB*	*qnrC*	*qnrD*	*qnrS*	*qepA*
**NB04V**	-	-	-	-	-	-
**NB08V**	-	-	-	-	-	-
**NB09V**	-	-	-	-	-	-
**NB10V**	-	-	-	-	-	-
**NB11V**	-	-	-	-	-	-
**NB12V**	-	-	-	-	-	-
**NB13V**	-	-	-	-	-	-
**NB14V**	-	-	-	-	-	-
**NB15V**	-	-	-	-	-	-
**NB16B**	-	-	-	-	-	-
**NB17V**	-	-	-	-	-	-
**NB18V**	-	-	-	-	-	-
**NB19V**	-	-	-	-	-	-
**NB20V**	-	-	-	-	-	-
**NB21V**	-	-	-	-	-	-
**NJ01V**	-	-	-	-	-	-
**NJ02V**	-	-	-	-	-	-
**NJ04V**	-	-	-	-	-	-
**NJ06V**	-	-	-	-	-	-
**NJ07V**	-	-	-	-	-	-

**NJ10V**	-	-	-	-	-	-
**NJ12V**	-	-	-	-	-	-
**NJ13V**	-	-	-	-	-	-
**NJ14V**	-	-	-	-	-	-
**NJ15V**	-	-	-	-	-	-
**NJ18V**	-	-	-	-	-	-
**NJ21V**	-	-	-	-	-	-
**NJ22V**	-	-	-	-	-	-
**NB01E**	-	-	-	-	-	-
**NB02E**	-	-	-	-	-	-
**NB03E**	-	-	-	-	-	-
**NB04E**	-	-	-	-	-	-
**NB05E**	-	-	-	-	-	-
**NJ01E**	-	-	-	-	-	-
**NJ02E**	-	-	-	-	-	-
**NJ03E**	-	-	-	-	-	-
**NJ04E**	-	-	-	-	-	-
**NJ05E**	-	-	-	-	-	-
**NJ08E**	-	-	-	-	-	-
**NJ09E**	-	-	-	-	-	-
**NJ10E**	-	-	-	-	-	-
**NJ11E**	-	-	-	-	-	-
**NJ12E**	-	-	-	-	-	-
**EB01V**	-	-	-	-	-	-
**EB02V**	-	-	-	-	-	-
**EB03V**	-	-	-	-	-	-
**EB04V**	-	-	-	-	-	-
**EB05V**	-	-	-	-	-	-
**EB06V**	-	-	-	-	-	-
**EB07V**	-	-	-	-	-	-
**EB08V**	-	-	-	-	-	-
**EB09V**	-	-	-	-	-	-
**EB10V**	-	-	-	-	-	-
**EB11V**	-	-	-	-	-	-
**EB12V**	-	-	-	-	-	-
**EB13V**	-	-	-	-	-	-
**EB14V**	-	-	-	-	-	-
**EB15V**	-	-	-	-	-	-
**EB01E**	-	-	-	-	-	-
**EB02E**	-	-	-	-	-	-

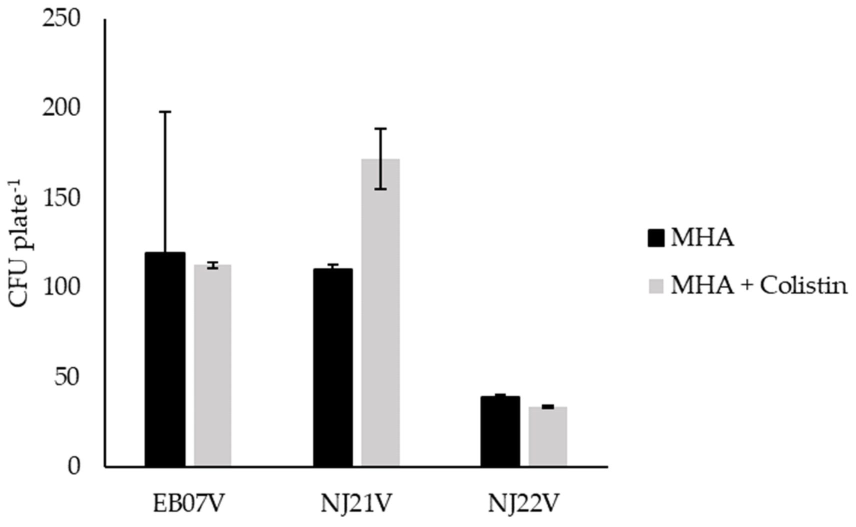

Figure S1. Plasmid curing of the isolates EB07V, NJ21V and NJ22V. The concentrations of ethidium bromide were: 0.05 g L^{-1} for EB07V, 0.025 g L^{-1} for NJ21V and NJ22V. The number of CFU was determined in MHA plates with and without colistin.
